# Bone regeneration with autologous adipose-derived mesenchymal stem cells: A reliable experimental model in rats

**DOI:** 10.1016/j.mex.2020.101137

**Published:** 2020-11-07

**Authors:** F. Javier Barrientos, Luis Miguel Redondo, Mercedes Alberca, Ana María Sánchez, Javier García-Sancho

**Affiliations:** a Servicio de Cirugía Maxilofacial, Hospital General de Castellón, Spain; b Servicio de Cirugía Maxilofacial, Hospital del Rio Hortega, Valladolid, Spain; c Citospin SL, Edificio I+D Campus Miguel Delibes, Valladolid, Spain; d Instituto de Biología y Genética Molecular (IBGM), Universidad de Valladolid y Centro Superior de Investigaciones Científicas (CSIC), Valladolid, Spain

**Keywords:** Adipose tissue, Animal model, Bone tissue engineering, Mesenchymal stem cell

## Abstract

The adult mesenchymal stem cell (MSC) has been proposed to be the definitive tool in regenerative medicine due to its multi-differentiation potential and expansion capacity *ex vivo*. The use of MSCs on bone regeneration has been assessed in several studies, obtaining promising results. However, the endless combinations that can be tested and the heterogeneity in the experimental conditions become a drawback when comparing results between authors. Moreover, it is very hard to find autologous studies using adipose-derived MSCs (AD-MSC) in rodents, which is the most used preclinical animal model.

In this article an experimental model for basic bone tissue engineering research is described and justified, on which adult AD-MSCs are safely isolated from the rat dorsal interscapular fat pad, allowing *ex vivo* expansion and autogenous orthotopic reimplantation in a bilateral mandibular bone defect made in the same animal.

This reliable and reproducible model provides a simple way to perform basic experimentation studies in a small animal model using autologous MSC for bone regeneration or cell therapy techniques prior to improve the research on large animal models.

• Predictable and safe harvest of adipose-derived MSC. No need of animal sacrifice.
• Allows for autologous studies with the most frequently used animal model: the rat. No need of allogeneic or human MSC use and, therefore, immunological concerns are avoided.
• Bilateral mandibular critical size defect to allow direct control/experimental comparison.

## Graphical abstract

**Figure:**
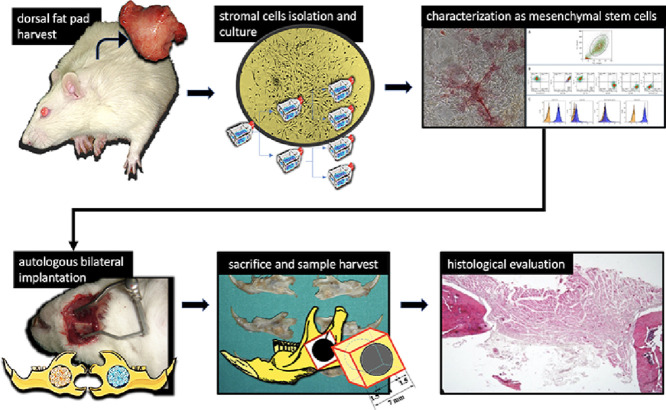

Specifications Table

**Table:** 

Subject Area:	Medicine and dentistry
More specific subject area:	*Bone regeneration with mesenchymal stem cells*
Method name:	*Autologous adipose-derived mesenchymal stem cells harvesting and implantation in a bilateral mandibular critizal-size defect model in rats*
Name and reference of original method:	Maglione M, Spano S, Ruaro ME, et al. In vivo evaluation of chitosan-glycerol gel scaffolds seeded with stem cells for full-thickness mandibular bone regeneration. J Oral Sci. 2017; 59(2): 225-232.
Resource availability:	N/A

## Rationale

The use of osteogenic cells on bone regeneration has been assessed in several studies obtaining promising results when adipose tissue mesenchymal stem cells (ATMSC) have been implanted with a variety of scaffolds [1], [2], [3], [4], [5]. However, direct comparison between studies is somehow impaired due to the heterogeneity in the experimental conditions [6], and sometimes it is not easy to determine which exact factor justifies a final result. Usually, tested MSCs are allogeneic or xenogeneic (human-derived), and sometimes the mandibular defect does not fulfill the critical size requirements or is unilateral. This variability precludes from advancing in bone tissue engineering (BTE) knowledge with reliability, from applying techniques in phylogenetically superior animals and from transferring the acquired knowledge to the clinical setting with predictability.

The selection of an appropriate animal model for a preclinical study is critical for the optimization and validation of the ATMSC therapeutic effects. A reproducible experimental model that can be imitated by other experimentation groups is desirable to compare different methodologies (biomaterials, cell therapy strategies, etc.) in a standardized context [7,8]. In the present study, a simple, safe and reliable animal experimental model is described and proposed as a standardizable method to test basic and preliminary autogenous BTE strategies.

## Methods

*This study complies with the ARRIVE guidelines and was carried out in accordance with the EU Directive 2010/63EU and national guidelines for the care and use of laboratory animals.*

## Experimental model

To illustrate the described methodology, 12 Adult male Wistar rats (300–400 gr. weight) were used. Animals were ear-marked for identification, housed in light, humidity and temperature-controlled facilities and given food and water ad libitum.

Two main groups of experiments on the same rat are made according to the defect filler: scaffold alone (control group, left hemimandible) and scaffold with autogenous ATMSC (experimental group, right hemimandible). Subgroups are made depending on the animal sacrifice and time of evaluation; in this case, 3 and 6 weeks were chosen.

## Obtaining the source of MSC

**Fig. 1:**
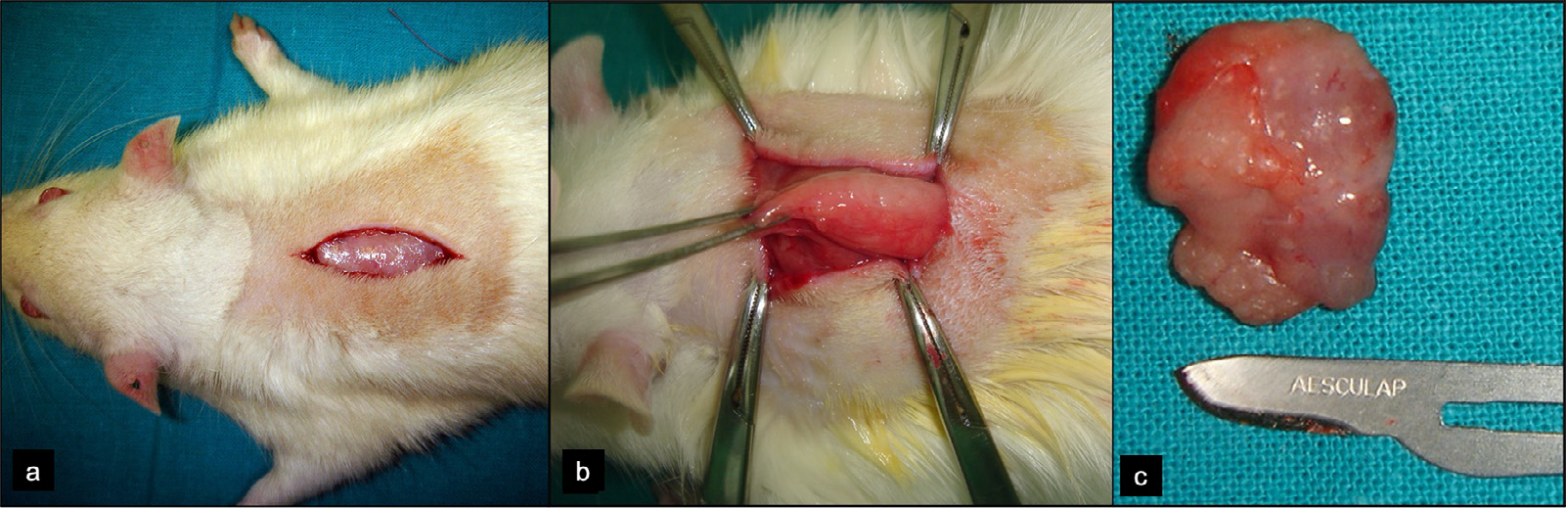
Surgical harvest of dorsal fat pad. (a) Midline vertical incision between both scapulae. (b) Access to the dorsal fat pad, which is easily identified as it is encapsulated. Gentle dissection without capsule disruption allows for simple removal. (c) Close view of the fat pad, showing an encapsulated adipose structure.

In a first surgical stage, surgical removal of each rat´s dorsal interscapular fat pad was performed; this procedure was made in prone position, after shaving and cleaning the upper back. For anesthesia, the animals were given an intraperitoneal injection of Ketamine (80 mg/kg) and local anesthetic (Ultracain^Ⓡ^, 0,5 ml without vasoconstriction) on the back. Vertical incision in the midpoint between the scapulae was made and, after blunt dissection of skin and subcutaneous flaps, access was gained to the fat pad (Fig. 1). Direct closure of the wound was made with monofilament 4/0.

## Isolation, culture and expansion of ATMSC populations

The fat pad was processed as described in the literature with light modifications so as to obtain putative ATMSC [9], [10], [11]. The fresh adipose tissue was embedded in HBSS (Hank ´s Balanced Salt Solution) and disaggregated. Fragments were placed in a conic tube and washed sequentially in PBS1x + glucose (5 mM) + BSA (2%) to induce the precipitation of the non-adipose phase. The supernatant adipose tissue was washed again for complete removal of hematic and local anesthetic components. The adipose phase was then digested (1 h, 37 °C) in 1 mg/ml Type I collagenase A (Roche Molecular Biochemicals) and neutralized afterwards. The cell phase was centrifuged (1200 rpm, 5 min.) to separate mature adipocytes and the stromal vascular fraction. Floating adipocytes were removed, and pelleted stromal cells were NH_4_Cl treated (160 mM, 5 min.) so as to lyse red blood cells. NH_4_Cl was neutralized with DMEM + 10% FBS + 1% de penicillin / streptomycin + 1% glutamine and a new centrifugation step was made (5 min, 2500 rpm). After supernatant removal, the cell pellet was passed through a 100 μm. cell strainer before plating and culturing at high density (20–30.000 cells/cm^2^) in a 200 μl. solution containing DMEM + 10% FBS + 1% Penicillin/Streptomycin + 1% glutamine (48 h, 37 °C, 5% CO_2_ atmosphere). Then, wash with FBS was made to remove the non-adherent cells. Cells were maintained for 5–7 days and the culture medium was subsequently changed every 2–3 days. Cells were grown to subconfluence (confluence was reached after 12–14 days) and passaged by standard methods of trypsinization.

**Fig. 2:**
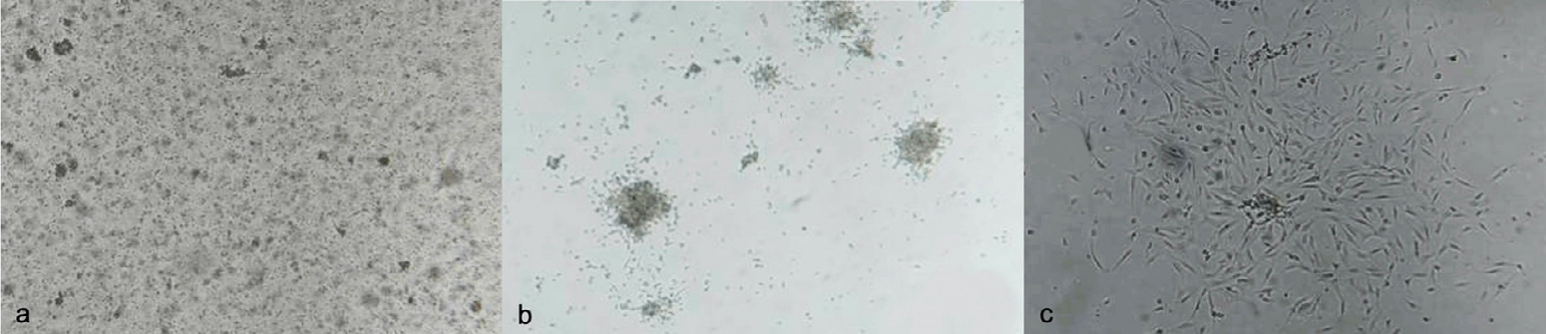
After 24 h of collagenase fat disaggregation and preincubation, an heterogenous conglomeration of cells was observed under light microscopy (a). After a 5–7 days preincubation period the non-adherent cell fraction was removed by means of thorough wash and culture medium renewal. At this stage, symmetric cell colonies (b) with homogeneous *fibroblastic-like* cell populations (c) were observed.

As a result, a population of mesenchymal cells was harvested from the dorsal fat pad of each one of the 12 rats (Fig. 2).

## In vitro essay

### Phenotype identification by flow cytometry

To confirm that the isolated stromal cells maintain their phenotypic characteristics after growth in culture, flow cytometry analysis was performed [12]. Passage 3 cells were harvested and suspended in PBS for flow cytometry at 1.10^6^ cells/ml concentration. After brief centrifugation, cells were resuspended in buffer (BD Biosciences) and a 100 μl. sample of cell suspension was incubated for 15 min at room temperature with fluorescein isothiocyanate conjugated CD90, phycoerythrin (PE)-conjugated CD31, phycoerythrin cyanine 5 (PC5)–conjugated CD45 and allophycocyanin-conjugated CD29 (Becton Dickinson Biosciences, San Jose, CA, USA) [13]. Flow cytometry was performed on a FACSCalibur^Ⓡ^ (BD Biosciences, Hospital Clínico de Salamanca, Servicio de Hematología unidad de Terapia Celular). The data were analyzed with Kaluza flow cytometer software (Beckman Coulter, Unidad de Terapia Celular del Instituto de Biología y Genética Molecular de Valladolid) [14]. Using forward and side scatter profile, debris and dead cells were discarded. CImmunophenotyping was performed with antibodies against mouse-rat antigens CD29-APC, D45-PerCP/Cy5.5 and CD90-FITC.

**Fig. 3:**
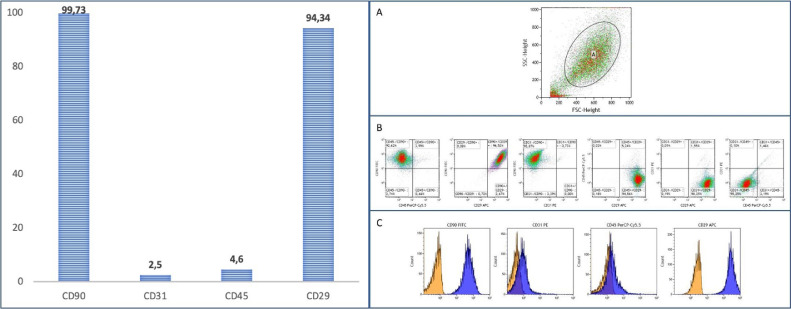
Flow cytometry. The bar graph (left) shows the percentage of positive cell surface markers of mesenchymal cells at passage 3. Cell characterization (right): A) Cell population. B) Cell surface markers in mesenchymal cells. C) Mean fluorescence intensity of stained mesenchymal cells (blue) and control non-stained mesenchymal cells (orange).

Harvested cells from fat samples could be expanded in all cases. All of them adhered to plastic surfaces, expressed antigens CD29, CD90 and were negative for CD31 and hematopoietic antigen CD45, as described [13,15] (Fig. 3).

### Multilineage differentiation potential

To comply with the MSC definition criteria, the multipotency capabilities of the isolated cells must be assessed, which is made by submitting them to differentiation towards the osteogenic, adipogenic and chondrogenic cell lines in vitro. A description of the multi-differentiation essay that was made in our study is presented below.

#### In vitro osteogenic differentiation

**Fig. 4:**
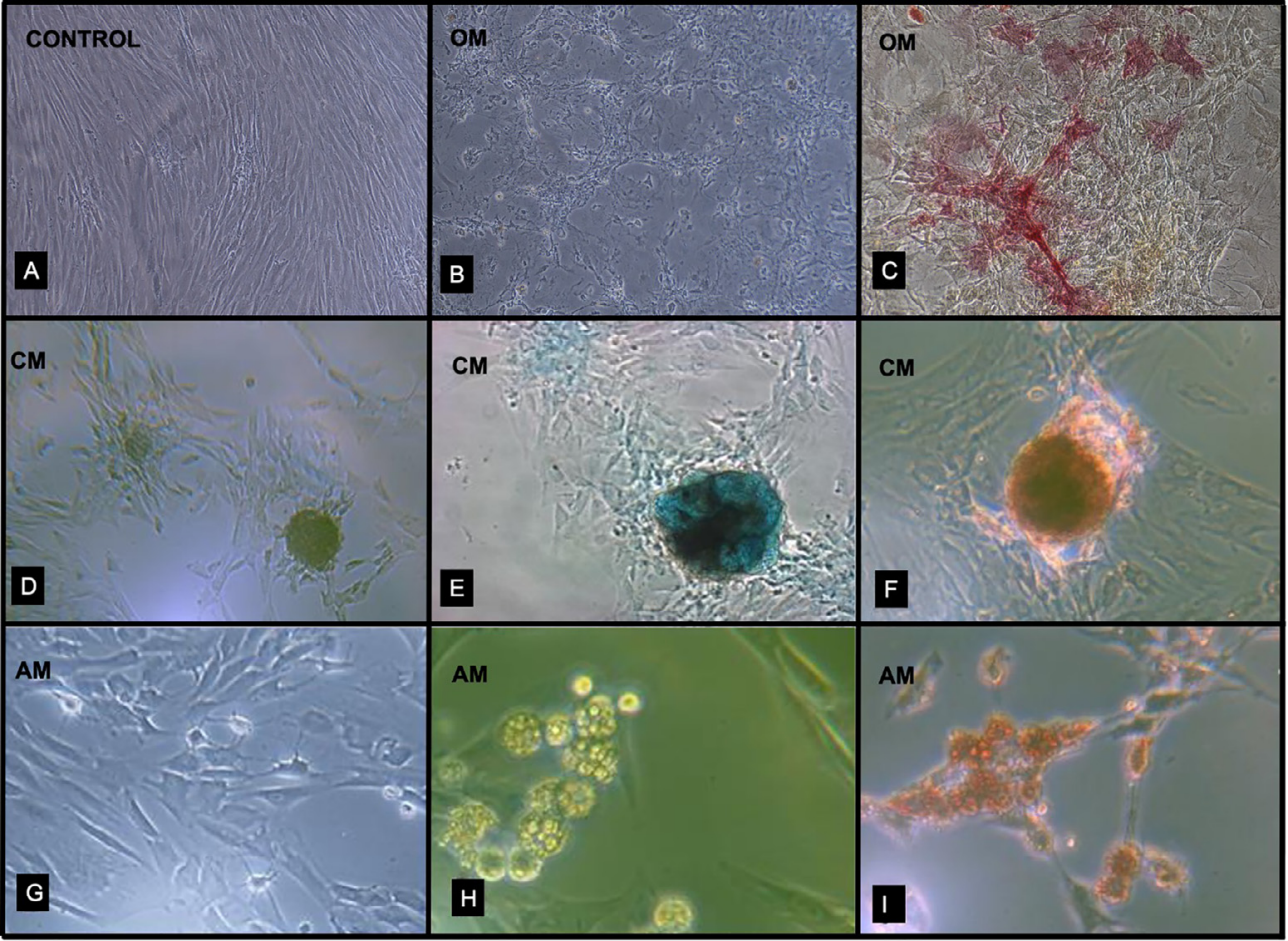
Multilineage differentiation essay. Mesenchymal cells grown in basal (control) culture medium did not differentiate and retained their characteristic spindle *fibroblastoid* morphology (A). Osteoblastic morphological changes (cuboidal cytoplasm with multiple cellular projections) after 16 days of incubation in Osteogenic Medium (OM) (B,10x). Osteogenic differentiation was demonstrated by red staining in the Fast Red test, involving phosphatase alkaline activity (C,10x). (D) Chondroblastic cell changes (10x) after 16 days of incubation in Chondrogenic Medium (CM), although cell shape began to transform into pellets at 7 days. Differentiation into chondrogenic lineage was demonstrated by Alcian Blue (E, 20X) and Safranin O staining (F, 20X). Morphological adipocyte-like changes after incubation in adipogenic medium (AM). Cell shape began to transform into ovoid morphology at 7 days of incubation (G, 10X). Lipid droplets could be identified early at the second week (H, 20x). Intracellular lipid droplets indicating adipogenic differentiation were confirmed by Oil Red O staining (I,10x).

To induce osteogenic differentiation, in our study, passage 3 cells (1.10^4^ cells/cm^2^) were seeded and cultured for 16 days onto 6-well tissue culture plates (BD Biosciences) with osteogenic medium (OM ) (ascorbic acid 60 μM. + dexamethasone 0.1 μM + glycerophosphate 10 mM (StemPro^Ⓡ^ Osteogenesis Differentiation kit (Gibco)). The medium was replaced twice a week. Qualitative phosphatase alkaline activity [9] was investigated with the Fast Red test (Sigma Fast^Ⓡ^, Sigma-Aldrich). Results in our study are shown in Figure 4A-C.

#### In vitro chondrogenic differentiation

Passage 3 cells (1.10^4^ cells/cm^2^) were cultured in chondrogenic medium (StemPro^Ⓡ^ Chondrogenesis Differentiation kit (Gibco)), which wasreplaced twice a week. After a 16 days culture period, the presence of chondrogenic pellets was investigated with Alcian Blue [16], [17], [18] and Safranin O staining (Sigma-Aldrich). Results are shown in Figure 4D-F.

#### In vitro adipogenic differentiation

Passage 3 cells (1.10^4^ cells/cm^2^) were cultured for 16 days onto tissue culture plates with adipogenic medium (StemPro^Ⓡ^ Adipogenesis Differentiation kit (Gibco)). The medium was replaced twice a week. Adipogenic differentiation was investigated by Oil Red O staining (0.5% oil red O in isopropanol) [16] (Sigma-Aldrich). Results are shown in Figure 4G-I.

### Biocompatibility cell-scaffold

ATMSCs and the chosen scaffold should be checked for biocompatibility prior to implantation. Thus, a preliminary 14 days coculture may be made; later, samples may be pipetted out and morphologically observed by scanning electron microscopy (SEM).

## Scaffold preparation and cell seeding

**Fig. 5:**
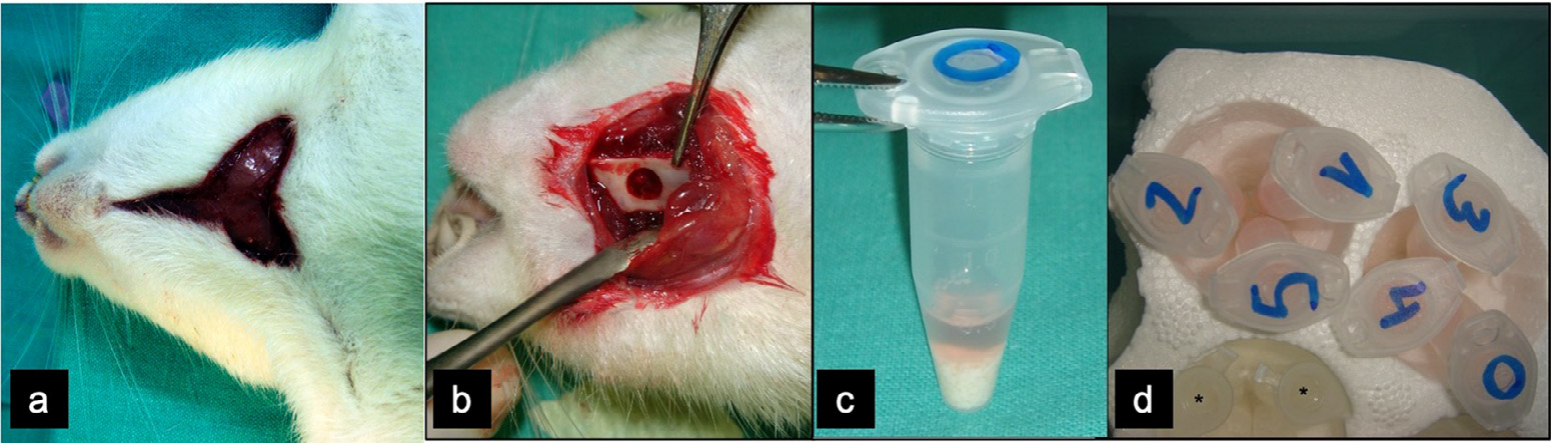
Second surgical stage. (a) Neck incision for bilateral mandibular angle approach. (b) A 4 mm. rounded bone defect made in the right (experimental) hemimandible to be filled with scaffold and ATMSC (c). The same but with scaffold alone must be done in the contralateral side. Each ATMSC container must be marked with each rat number to ensure autologous implantation (d).

Cells were trypsinized and lifted off culture dishes upon subconfluence, washed and quantified. Two sterilized recipients with porous scaffold were prepared for each animal; one of them was submerged in autologous 2.10^6^ MSC suspension (Fig. 5). The scaffold can optionally be pre-coated by immersion into a 100 mg/ml. solution of fibronectin (Collaborative Biomedical Products; Collaborative Research Inc., Bedford, MA) and subjected to 1 h incubation at 4 °C so as to improve cell adherence. Negative pressure may be gently applied into a capped syringe containing cells and scaffold so as to promote the evacuation of the air bubbles out of the inner pores and the replacement of these pores by cells [19]. Cells were allowed to adhere for 2 h and implanted into animals as follows.

## In vivo implantation

In a second surgical step, rats were anesthetized in a similar manner and operated in supine position. The surgical site (i.e. the neck) was cleaned with Betadine and bilateral submandibular approaches joining in the midline were made; flaps were raised showing the masseter muscle on which surface the facial nerve can be identified and therefore preserved.

The lower edge of the mandible was identified by palpation and direct incision was made over it. Subperiosteal dissection was carried on so as to expose the mandibular angle and ramus. The periosteum was removed and a 4 mm. diameter rounded defect was made on each hemimandible with a 4 mm. electric carbon burr under continuous physiologic serum irrigation. Caution was taken so as not to disrupt the lingual side tissues, leading to an orocervical fistula. The defects were washed to remove bone remnants and filled with the scaffold with/without cells according to the planned group distribution (Fig. 5). The skin was sutured and the animal was clinically monitored.

## Clinical follow-up, animal sacrifice and sample harvest

In the postoperative period, general animal condition was carefully monitored and, the surgical sites, closely inspected for potential complications. The postoperative period following both operations was uneventful and every animal recovered well with no signs of infection or other anomalies despite some soft tissue paramandibular swelling after ostectomy, which resolved spontaneously.

**Fig. 6:**
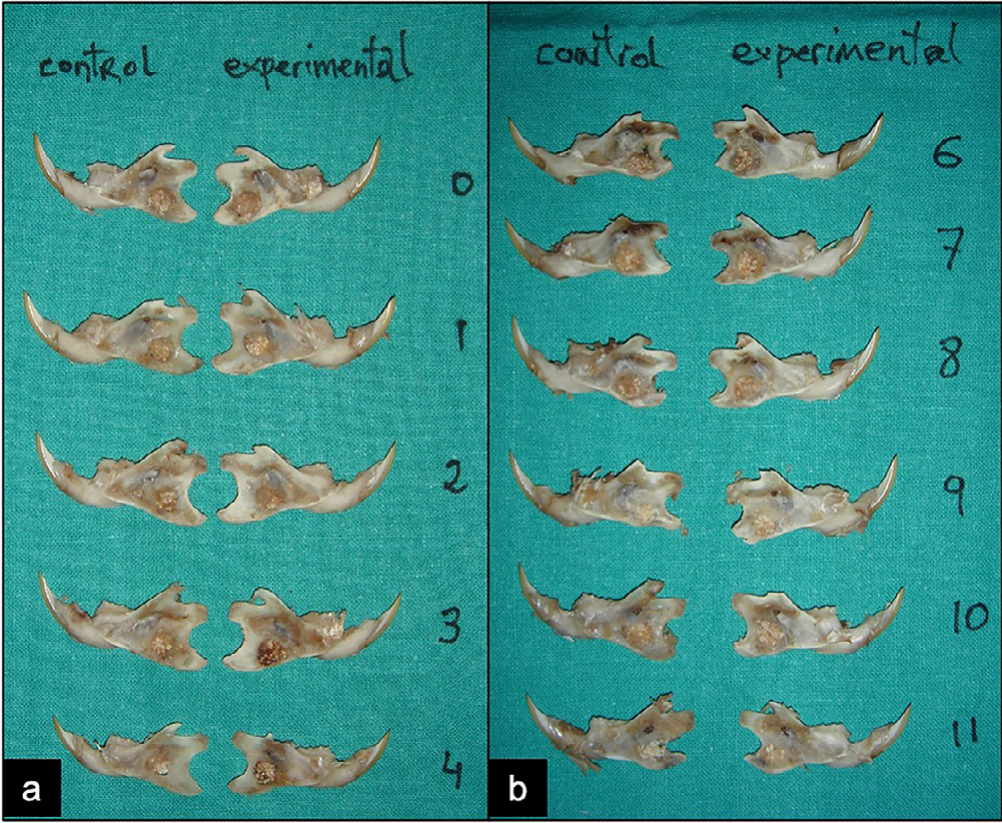
Specimens ready for evaluation (a: 3 weeks; b: 6 weeks). Both hemimandibles of each animal are used, allowing direct comparison. In our study, specimens in rat number 5 were discarded as the defect filler was accidentally pulled out in one hemimandible during specimen preparation and soft tissue removal.

**Fig. 7:**
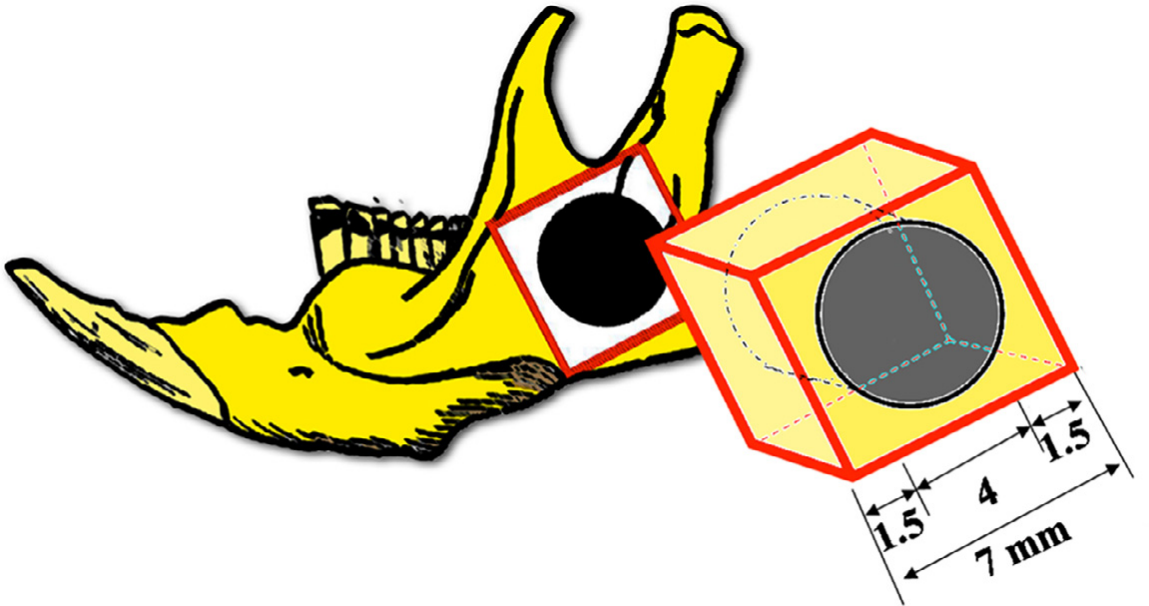
A square block is excised from each hemimandible including the bone defect with a 1.5 mm. bone margin.

Once the experimental periods conclude (3 and 6 weeks after implantation) the animals were killed by intraperitoneal anesthetic overdose injection of ketamine (Imalgene 500^Ⓡ^). Specimens were obtained (Fig. 6) by mandible symphysis bisection, dislocation and block hemimandible excision. They were fixed in 10% formalin (24 h, 4 °C) and macroscopically evaluated considering the following parameters: a) anatomic and tissue organization of the defect; b) infections, exudates or tissue collections; c) biomaterial displacement or extrusion; d) fractures; e) dental degenerative changes; f) bone sequestration and g) consistency and morphology of the defect. Samples including the bone defect were excised from each mandible (Fig. 7). These blocks were left for overnight decalcification (SurgipathⓇ Decalcifier I (Surgipath Medical Industries, Inc.)) and then trimmed, processed and embedded in paraffin wax, sliced with a rotation microtome and stained with hematoxylin-eosin for histological evaluation and statistical analysis.

## Method validation

The rodent animal model for bone regeneration study in the craniofacial area is widely supported in the scientific literature [20]. Results in ectopic bone regeneration models with MSC in rodents are somewhat predisposed due to the absence of biomechanical forces acting on the bone forming tissue. Moreover, biological environment and, therefore, vascularization found by a small cell-scaffold complex at a subcutaneous level is more favorable for diffusion, allowing an optimal supply of oxygen and nutrients for MSC. Bone neoformation observed in unfavorable microenvironment has a greater value [21,22]. Thus, an orthotopic model represents a more reliable bone healing simulation because of bone microenvironment around the complex.

Critical size defects are invaded by fibrous scar tissue, preventing bone regeneration. The surgical defect used by some authors [24], [25], [26] and adopted in this experimental study is a bilateral critical size (i.e. 4 mm. diameter) circular defect in the center of the mandibular angle, whereas others prefer a small defect in the mandibular margin [27,28]. Surprisingly, some authors [27], [28], [29] perform unilateral defects in the mandible claiming to preserve masticatory function. The safety of bilateral mandibular defects in rats has been extensively proven and allows to make direct comparisons in the same animal while duplicating the sample size. Therefore, the number of involved rats can be adjusted to the strictly necessary, which fulfills the ethical requirements concerning animal experimentation.

Other common model in rats is the craniotomy defectbut, when comparing studies, it should be noted that some authors do not fulfill the critical size defect conditions, which must be 8 mm. in the rat´s cranium; besides, it is not subjected to biomechanical forces and may compromise anatomical structures like the duramater [23,^30^,31]. The segmental mandibular defect, the mandibular distraction model, the spinal fusion model and femur bone defects are also frequently used [32], [33], [34], but require stabilization devices which add technical difficulty, economic cost and morbidity [21,32] leading to discard some animals and to reduce the sample size. Mandibular dimensions in the adult rat enable to perform this defect with low risk of iatrogenic fractures [35] and avoid the use of mechanical stabilization devices. A simple surgical technique involves better reproducibility and helps reducing costs while allowing a larger sample and a more accurate interpretation of the results.

Whereas the MSC donor source, initially it was thought that the low immunogenicity [36], [37], [38], [39], [40] and immunosuppressive capacity of MSCs would allow transplantation into immunocompetent recipients with no immunosuppressants. Despite this, some observations in the literature suggest prudent behavior when considering allo or xenotransplants, which in turn are the most commonly used [25,^26^,^29^,^30^,^34^,40]. Though MSCs inhibit the proliferation of allo-activated lymphocytes, they also release factors that can activate the rest of immune cells; allogeneic MSCs may be lysed by CD8 T and NK cells and, autologous MSC, by NK cells [41], [42], [43]. This NK-mediated lysis is inversely correlated with the expression of HLA class I by MSCs; undifferentiated ATMSCs are negative for markers such as HLA-ABC and HLA-DR but, after differentiation, they express HLA-DR [36]. Therefore, in an allo or xenogeneic transplant, there is a risk of rejection or allogeneic sensitization and may require the use of immunosuppressants such as FK 506 or athymic animals (without cellular immunity) [25,^26^,^42^,[44], [45], [46]]. Immunocompromised animal models have certain limitations as these animals are more prone to complications such as infections and systemic diseases and have an undesirable microenvironment for regeneration due to the modification of the normal balance between cells that are involved in the healing process. This alters the levels of growth factors and the inflammatory response by the host macrophages and other immune cells. Immunocompetent animal models avoid these drawbacks and allow a better simulation of the physiological and biological environment [26,^47^,48]. With this controversy in mind, an autogenous transplant appears to be a better alternative in order to overcome immunological issues, at least at a basic experimental level. Our results show no bone resorption,abnormal bone callus formation,infections or severe inflammatory reactions, therefore suggesting the feasibility of autologous ATMSC in this kind of application.

Autologous BMMSC transplant in small animals like rats is not feasible given that it requires harvesting a long bone and extracting the bone marrow contents for MSC isolation. This involves subsequent animal sacrifice for ethical reasons [49], [50], [51]. Since Zuk´s reports [9], attention has been focused on adipose tissue as a reliable alternative stromal source of authentic MSC with certain advantages like cell availability per mm^3^ of tissue and the easy and safe harvest, which allows to plan an autologous MSC orthotopic study in a small animal model, though it was considered not feasible [51].

When it comes to adipose tissue sources, rats have a small subcutaneous adipose compartment and large dissection areas would be required for significant tissue harvest. Alternative sources include cervical (brown or white), visceral (gonadal, perigastric, perirenal and perintestinal) or inguinal fat [14,^34^,47]. Obtaining a clinically relevant cell quantity demands an excessive fat removal, which is very disabling for the animal and, subsequently, sacrifice is necessary [52]. In this model we demonstrate that interscapular brown fat pad in an adult rat can be easily and safely harvested [27,^53^,54], providing significant tissue amount for ATMSC isolation. In lower weight rats, several fat pads might have been necessary for successful ATMSC isolation, precluding an autologous study. No relevant morbidity may be expected from brown fat pad removal [27], as seen in our study, though brown adipose tissue belongs to the rodent´s immunoneuroendocrine system (it is a natural thymus antagonist and is involved in temperature regulation) [55] and its removal modifies cellular immunity increasing the risk of developing autoimmune diseases [53]. In a study by Kurzyk [15], who compared several fat sources in rats, the inguinal area yielded the highest raw adipose tissue amount and the gonadal area provided the highest amount of stromal vascular fractions (SVF). However, interestingly, the cervical dorsal brown tissue was the best source when considering the number of SVFs per gram of adipose tissue. Moreover, cells obtained from dorsal fat pad showed the highest proliferative potential.

Subcutaneous and visceral adipose tissue not only differ in anatomical location but also in metabolic and growth rates [56], and even distant visceral adipose tissue sources may yield different MSC counts [51]; in the same way, ATMSC obtained from different anatomical locations show differences in terms of proliferation and differentiation probably due to the existence of ATMSC subpopulations. According to Arrigoni [51], the anatomical location seems to have minor effects on the features of rat´s ATMSCs but this is an important point to be elucidated in humans so as to optimize the results in the clinical setting.

## Declaration of Competing Interest

'The Authors confirm that there are no conflicts of interest.
